# Triptolide Inhibits Osteoclast Differentiation and Bone Resorption* In Vitro* via Enhancing the Production of IL-10 and TGF-*β*1 by Regulatory T Cells

**DOI:** 10.1155/2016/8048170

**Published:** 2016-06-19

**Authors:** Huihui Xu, Hongyan Zhao, Cheng Lu, Qi Qiu, Gui Wang, Jing Huang, Minghui Guo, Baosheng Guo, Yong Tan, Cheng Xiao

**Affiliations:** ^1^Beijing University of Chinese Medicine, Beijing 100029, China; ^2^Institute of Clinical Medicine, China-Japan Friendship Hospital, Beijing 100029, China; ^3^Institute of Basic Theory of Chinese Medicine, China Academy of Chinese Medical Science, Beijing 100700, China; ^4^Institute of Basic Research in Clinical Medicine, China Academy of Chinese Medical Science, Beijing 100700, China; ^5^Institute of Clinical Pharmacology, Beijing Anzhen Hospital, Capital Medical University, Beijing 100029, China; ^6^Institute for Advancing Translational Medicine in Bone & Joint Diseases, School of Chinese Medicine, Hong Kong Baptist University, Kowloon Tong, Hong Kong

## Abstract

Triptolide, a purified component of* Tripterygiumwilfordii Hook F*, has been shown to have immunosuppressive and anti-inflammatory properties in rheumatoid arthritis (RA). Although triptolide has demonstrated that it could suppress bone destruction in collagen-induced mice, its therapeutic mechanism remains unclear. Many studies have investigated the effect of triptolide on Tregs and Tregs-related cytokine involved in RA. Additionally, previous studies have implied that Tregs inhibit osteoclast differentiation and bone resorption. Thus, in this study we aimed to explore the regulatory mechanism by which triptolide influences the Treg-mediated production of IL-10 and TGF-*β*1 to affect osteoclast differentiation and bone resorption. In cocultures system of Tregs and mouse bone marrow macrophages (BMMs), Tregs inhibited the differentiation of osteoclasts and reduced the resorbed areas significantly and the production of both IL-10 and TGF-*β*1 was upregulated. When the coculture systems were pretreated with triptolide, they produced higher levels of IL-10 and TGF-*β*1. Our data indicate that triptolide enhances the suppressive effects of Tregs on osteoclast differentiation and bone resorption by enhancing the secretion of IL-10 and TGF-*β*1. Tregs are most likely involved in the triptolide-mediated regulation of bone metabolism and may provide a potential therapeutic target for the treatment of inflammatory bone destruction.

## 1. Introduction

*Tripterygiumwilfordii Hook F *(TWHF) extracts have been widely used in the treatment of autoimmune and inflammatory diseases, including rheumatoid arthritis (RA). Triptolide, the major component of TWHF, has many beneficial functions including immunosuppression, anti-inflammation, and anticarcinogenesis [1, 2]. RA is a chronic autoimmune disease characterized by chronic synovial inflammation that results in the progressive destruction of bone. Bone destruction in RA is caused in part by the activation of osteoclasts. Osteoclasts are multinucleated cells (MNCs) that are differentiated from the mononuclear cells of the monocyte and macrophage lineages. Pivotal to this process is the receptor activator of nuclear factor-*κ*B ligand (RANKL) and the macrophage colony-stimulating factor (M-CSF), both of which are key cytokines for osteoclast differentiation, survival, and function [3]. There is accumulating evidence that osteoclasts are major cell type responsible for the bone destruction of RA [4, 5]. Previous data have shown that triptolide may partially attenuate RA by preventing bone destruction and may inhibit osteoclast formation [6]. In addition, most of the cytokines that are involved in regulating osteoclast differentiation, such as tumor necrosis factor-*α* (TNF-*α*), interferons (IFNs), and interleukins (ILs) [7, 8], also affect the immune system, suggesting the existence of a relationship between these two systems.

CD4^+^CD25^+^Foxp3^+^ T cells, termed regulatory T cells (Tregs), play a critical role in limiting autoimmune responses by modulating the adaptive and innate immune systems. It was reported that triptolide can regulate Tregs and modulate the production of cytokines such as interleukin-10 (IL-10) and transforming growth factor-*β* (TGF-*β*)* in vivo *[9]. Triptolide is the active ingredient of T2, a chloroform/methanol extract of TWHF. Li et al. found that T2 enhances the level of Tregs* in situ* and modulates the production of inflammatory cytokines such as IL-10 and TGF-*β* [10]. Other previous studies showed that Tregs inhibit osteoclast differentiation and function in a cytokine-dependent manner [11]. Tregs secrete cytokines such as IL-10 and TGF-*β*1 that appear to play a key role in suppressing osteoclast differentiation and bone resorption [12]. However, little information is available regarding the role of triptolide in the regulation of osteoclastogenesis by Tregs.

Therefore, the aims of our study were to investigate the effect of triptolide on the Treg-mediated regulation of osteoclast differentiation and bone resorption and to determine the mechanism by which this regulation occurs.

## 2. Materials and Methods

### 2.1. Animals and Drug

Male C57BL/6 mice that were 6-7 weeks old and weighed 20–22 g were purchased from the Research Institute of Experimental Animals, Chinese Academy of Medical Science. They were housed in specific-pathogen-free environment (22 ± 1°C, 12 h light/dark cycle) with free access to a standard mouse diet and water. The experiments were approved by the Animal Care & Welfare Committee of China-Japan Friendship Hospital. Triptolide (Sigma-Aldrich, St. Louis, MO, USA) was solubilized in dimethyl sulfoxide (DMSO) and diluted in phosphate buffered saline before usage.

### 2.2. Osteoclast Differentiation

Bone marrow cells were isolated from the bilateral tibias and femurs of C57BL/6 mice. After the connective tissues were removed from the bones, the double ends of the bones were cut off and the bone marrow was then flushed out. The red blood cells were removed using red blood cell lysis solution (Solarbio, Beijing, China) and suspended in Alpha Modified Eagles Medium (*α*-MEM) (Hyclone, Logan, USA) containing recombinant murine M-CSF (20 ng/mL) (PeproTech, Rockey Hill, USA), 10% fetal bovine serum (PAN Biotech GmbH, Aidenbach, Germany), and 1% antibiotics (100 U/mL of penicillin A and 100 U/mL 100 *μ*g/mL of streptomycin) (Gibco BRL, Grand Island, NY, USA). The cells were cultured in a humidified atmosphere of 5% CO_2_ at 37°C. After 24 hours, the nonadherent cells were collected and seeded in 24-well culture plates at density of 5 × 10^5^ cells/well in *α*-MEM supplemented with 10% FBS and M-CSF (20 ng/mL). After 3 days, the bone marrow macrophages (BMMs) that remained on the bottom of the wells and became adherent cells were used for osteoclastogenesis. After the removal of the culture medium, the BMMs were cultured for another 5 days in *α*-MEM containing 20 ng/mL M-CSF and 100 ng/mL recombinant murine soluble RANK ligand (RANKL) (PeproTech, Rockey Hill, USA). The culture medium was exchanged for fresh medium every 2 days to maintain a constant concentration of M-CSF and RANKL.

#### 2.2.1. Tartrate-Resistant Acid Phosphatase (TRAP) Staining and Osteoclast Activity Assay

After 5 days in culture, the cells were subjected to TRAP staining to determine OC number. The TRAP staining kit was purchased from Sigma-Aldrich (St. Louis, MO, USA). TRAP staining was performed according to the manufacturer's protocol. The cells were fixed and washed in triplicate and then incubated for 1 h at 37°C with TRAP staining solution. The TRAP-positive MNCs were visualized using a microscope. Those with 3 or more nuclei were scored and counted as osteoclast cells.

To measure the TRAP activity, the cells were treated with 50 *μ*L of phosphatase substrate solution (pH 5.0) containing 5 mM* p*-nitrophenyl phosphate (Sigma-Aldrich, St. Louis, MO, USA), 100 mM sodium citrate, and 50 mM sodium tartrate. After shaking for 10 min and incubating at 37°C for 1 h, the reaction mixtures were transferred to a new plate, and 50 *μ*L of 0.1 M NaOH was added to stop the reaction. The optical densities were read by a microplate spectrophotometer at 405 nm.

#### 2.2.2. Bone Resorption Pit Formation Assay

Bone slices (Immunodiagnostic Systems Limited, Boldon, UK) were put into 24-well culture plates, and nonadherent BMMs were plated on top of them. The cells were incubated with M-CSF (20 ng/mL) for 3 days before they were stimulated with RANKL (100 ng/mL) and various doses of triptolide. The culture medium was exchanged for fresh medium every 2 days in order to maintain a constant concentration of M-CSF and RANKL. After culturing the cells for 12 days at 37°C in a 5% CO_2_ incubator, the cells were removed from the bone slices by sonication in the presence of 0.25 M NH_4_OH, and then the bone slices were gradient dehydrated in 40%, 75%, 95%, and 100% ethanol and stained with 1% toluidine blue (Sigma-Aldrich, St. Louis, MO, USA) for 5 minutes. Finally, the areas of the bone resorption pits were calculated using the Leica Qwin image analysis software (Leica Microsystem, Germany).

### 2.3. Isolation and Purification of CD4^+^CD25^+^ Regulatory T Cells

Pooled cell suspensions were obtained from the spleens and lymph nodes (LNs) of C57BL/6 mice. CD4^+^ T cells were isolated from the spleens and lymph nodes and negatively selected using the EasySep*™* Mouse CD4 Positive T Cell Pre-Enrichment Kit (STEMCELL Technologies Inc., Vancouver, Canada). CD4^+^CD25^−^ regulatory T cells were then purified by negative selection using the EasySep® Mouse CD25 Positive Selection Kit (STEMCELL Technologies Inc., Vancouver, Canada).

The freshly isolated CD4^+^CD25^−^ T cells were plated at a concentration of 1 × 10^5^ cells/well in a 96-well plate that had been precoated at 37°C with anti-CD3 (10 *μ*g/mL) (BD Biosciences, San Jose, CA, USA) for 2 h. The cells were cultured in RPMI-1640 (Hyclone, Logan, USA) supplemented with anti-CD28 (2 *μ*g/mL) (BD Biosciences, San Jose, CA, USA), recombinant murine IL-2 (1000 U/mL) (PeproTech, Rockey Hill, USA), TGF-*β* (10 ng/mL) (R&D systems, Minneapolis, USA), 10% FBS, 100 U/mL penicillin, and 100 mg/mL streptomycin. After the cells were induced for 7 days, the T cells were purified again by positive selection using the EasySep Mouse CD25 Positive Selection Kit. Foxp3 expression was determined by flow cytometry.

Flow cytometry was applied for the identification of Treg. Anti-mouse CD4-FITC, anti-mouse CD25-APC, and anti-mouse Foxp3-PE were purchased from BD Biosciences (San Jose, CA, USA). The cells (approximately 1 × 10^6^) were first incubated with anti-mouse CD4-FITC and anti-mouse CD25-APC in the dark at 4°C for 30 minutes to stain the surface markers. Following fixation and permeabilization for intracellular staining, the cells were washed twice and stained with anti-mouse Foxp3-PE or an isotype control for another 30 minutes in the dark at 4°C; the cells were suspended in flow cytometry staining buffer and analyzed on a flow cytometer.

### 2.4. The Cytotoxicity of Triptolide against BMMs and Tregs

The CCK-8 assay was carried out according to the manufacturer's protocol to measure the effect of triptolide on cell viability. BMMs were seeded in 96-well plates at densities of 4 × 10^4^ cells/well and then preincubated with M-CSF (20 ng/mL) for 3 days before stimulation with RANKL (100 ng/mL) and triptolide (0, 2.5, 5, 10, 20, or 40 nM). After 5 days incubation, cells were added with 10 *μ*L CCK-8 and incubated for 3 hours at 37°C to measure the cytotoxicity against osteoclasts. Tregs were seeded in 96-well plates at densities of 1 × 10^5^ cells/well in the presence of various concentrations of triptolide. After 24 hours of incubation, cells were added with 10 *μ*L CCK-8 to measure the cytotoxicity of triptolide against Tregs. Both control groups were treated with vehicle (DMSO), respectively. The absorbance was measured at a wavelength of 450 nm using a microplate spectrophotometer.

### 2.5. Coculture of BMMs with Tregs

In the cell contact wells, isolated nonadherent BMMs (5 × 10^5^/well) were incubated with M-CSF (20 ng/mL) for 3 days and the Tregs were isolated as described above. Then the Tregs and BMMs were cocultured in 24-well plates with different Tregs/BMMs ratios (0 : 50, 1 : 50, 2 : 50, or 10 : 50) in *α*-MEM containing 10% FBS, 1% antibiotics, M-CSF (20 ng/mL), and RANKL (100 ng/mL) for another 5 days, with half of the medium being replaced every 2 days.

Transwell experiments were conducted in 24-well plates by culturing BMMs (5 × 10^5^/well) in the lower well and the Tregs (1 × 10^4^, 2 × 10^4^, or 1 × 10^5^) in the inserts. These cells were cocultured in the same medium and same Tregs/BMMs ratios as described for the cell contact wells. Half of the medium was replaced every 2 days.

After the incubation of 24 h, the supernatants in the cell contact groups and Transwell groups were collected and stored at −80°C. After being cocultured for 5 days, the cells were stained using TRAP to determine the number of osteoclasts, which were defined as TRAP-positive MNCs containing 3 or more nuclei. After 12 days, the bone slices were stained with toluidine blue and calculated by Leica Qwin image analysis software.

### 2.6. Effect of Triptolide on the Coculture of BMMs with Tregs

A coculture consisting of a 2 : 50 ratio of Tregs/BMMs was treated with triptolide for 5 days with M-CSF (20 ng/mL) and RANKL (100 ng/mL). Unless otherwise stated, neutralizing antibodies against IL-10 (10 *μ*g/mL) (PeproTech, Rocky Hill, USA) and TGF-*β*1 (10 *μ*g/mL) (BioLegend, San Diego, CA) were added in the coculture system. They are grouped as follows: BMMs, Tregs+BMMs, Tregs+BMMs+anti-IL-10+anti-TGF-*β*1, TP+BMMs, TP+Tregs+BMMs, and TP+Tregs+BMMs+anti-IL-10+anti-TGF-*β*1. After 24 h, the supernatant of each group was harvested to measure the levels of IL-10 and TGF-*β*1. The Mouse TGF-*β*1 Platinum and Mouse IL-10 Platinum ELISA Kits were purchased from eBioscience (San Diego, USA). All of the procedures were carried out according to the manufacturer's instructions.

Every 2 days, half of the medium was refreshed and kept a constant concentration of M-CSF, RANKL, and triptolide. Next, cells were stained for TRAP after being cocultured for 5 days. The bone slices were stained for toluidine blue to calculate the resorption pit after being cocultured for 12 days. A culture of BMMs alone treated with vehicle (DMSO) served as a control group.

### 2.7. Statistical Analysis

All of the data were expressed as means ± standard deviation (SD) and analyzed with the SPSS 20.0 program using one-way ANOVA followed by LSD test. Differences were considered to be statistically significant if the *P* values were less than 0.05 (^*∗*^ *P* < 0.05 and ^*∗∗*^ *P* < 0.01).

## 3. Results

### 3.1. Isolation and Identification of Tregs

**Figure 1:**
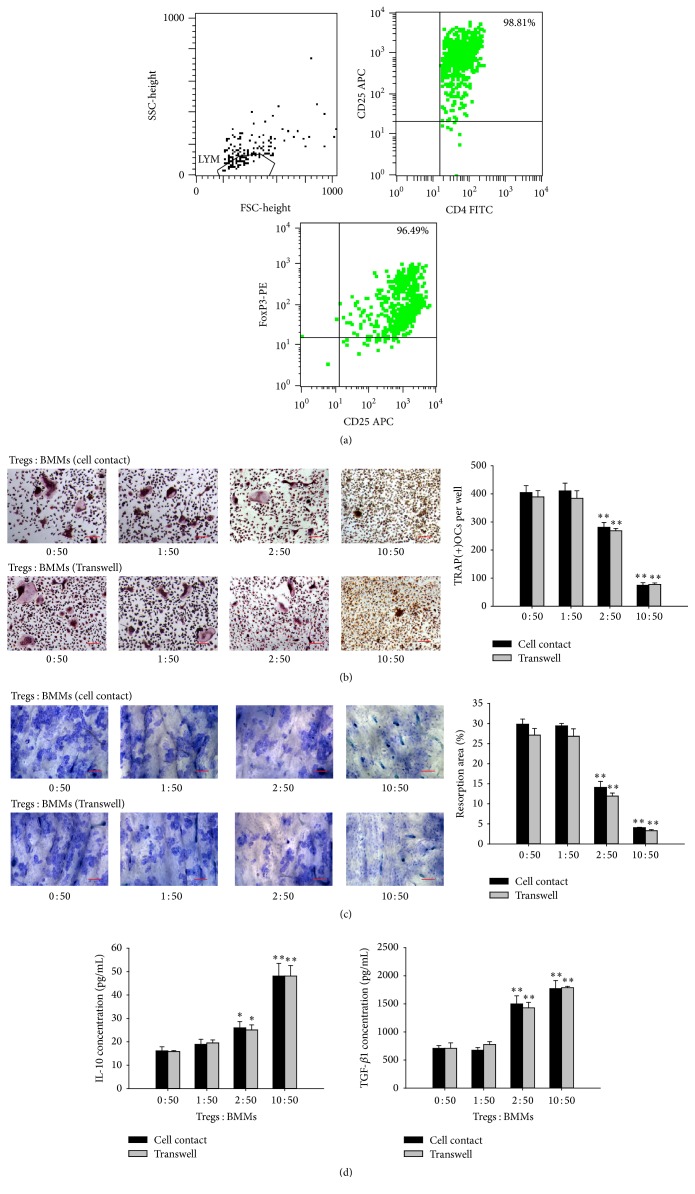
The cocultures of activited Tregs and BMMs. Activated Tregs and BMMs were cocultured for 5 or 12 days at different ratios in either cell contact wells or Transwells in the presence of 20 ng/mL M-CSF and 100 ng/mL RANKL. (a) The purification of Tregs was assessed by flow cytometry. (b) The cells were stained with TRAP, and the TRAP-positive cells containing 3 or more nuclei were scored. Scale bars = 100 *μ*m. (c) The area of the bone resorption pits of the osteoclasts was visualized by toluidine blue staining and measured using an image analysis program. Scale bars = 100 *μ*m. (d) The IL-10 and TGF-*β*1 levels in the coculture supernatants were measured by ELISA. ^*∗*^ *P* < 0.05 and ^*∗∗*^ *P* < 0.01 compared with the control groups (BMM alone groups).

CD4^+^ T cells were purified from mouse spleens and LNs, and CD4^+^CD25^−^ T cells were further negatively selected for induction. We stimulated CD4^+^CD25^−^ T cells with anti-CD3 (10 *μ*g/mL) and anti-CD28 in the presence of TGF-*β* and IL-2. Finally, flow cytometry revealed that the CD4^+^CD25^+^ T cells were 98.81% pure and the CD25^+^Foxp3^+^ T cells were 96.49% pure (Figure 1(a)).

### 3.2. Inhibitory Effects of Tregs on OC Differentiation and Bone Resorption

Treg-mediated suppression of osteoclasts depends on the activation of Tregs. To gain insights into the roles of Tregs on osteoclastogenesis, we used activated Tregs cocultured with BMMs at different ratios (0 : 50, 1 : 50, 2 : 50, or 10 : 50) to establish coculture systems. When the ratio of Tregs : BMMs was 1 : 50, no significant difference was observed on osteoclast differentiation and bone resorption. However, when the ratio approached 10 : 50, osteoclast formation and the absorbed surfaces of the bone slices were rarely observed. Thus, the coculture system comprising Tregs and BMMs resulted in a significant dose-dependent reduction in both TRAP-positive MNCs and the resorption area. We found that the number of TRAP-positive MNCs was significantly decreased in the coculture systems (the ratio of Tregs : BMMs was 2 : 50) compared with the cultures of BMMs alone, indicating that Tregs suppress osteoclast differentiation and osteoclastogenesis (Figure 1(b)). To examine the effect of Tregs on the function of osteoclasts, we measured the lacunar formation of the bone slices in the coculture groups as well as in the BMMs only group. We observed that the resorption area of the coculture groups showed a significant reduction (Figure 1(c)). These results indicate that regulatory T cells efficiently reduce the differentiation and function of osteoclast.

This coculture experiment was then modified to determine whether the Treg-mediated inhibition of osteoclast differentiation required direct cell-to-cell contact or soluble factors were required, we cocultured Tregs and BMMs in Transwell using the same ratios described above. Tregs inhibited osteoclast differentiation, not only in the direct coculture but also in the Transwell coculture system. No significant changes in either osteoclast count or resorption pit area were observed when direct cell-to-cell contact was prohibited (Figures 1(b) and 1(c)). These results suggest that Tregs inhibit osteoclast differentiation and bone resorption in a cytokine-dependent manner.

### 3.3. The Effect of Treg-Secreted IL-10 and TGF-*β*1 on OC Differentiation and Bone Resorption

To address the inhibitory mechanism of Tregs on osteoclastogenesis, we explored the expression level of IL-10 and TGF-*β*1 which were both anti-inflammatory cytokines mainly secreted by Tregs and linked to Treg expansion and/or functions [13]. We measured the level of IL-10 and TGF-*β*1 in the supernatants of cocultured cells grown in the presence or absence of a Transwell insert. The production of IL-10 and TGF-*β*1 was upregulated as the ratio of Tregs increased in both of the coculture systems. There was no significant difference between the cell contact and Transwell groups (Figure 1(d)).

### 3.4. Cytotoxicity of Triptolide against Tregs and Osteoclasts

**Figure 2:**
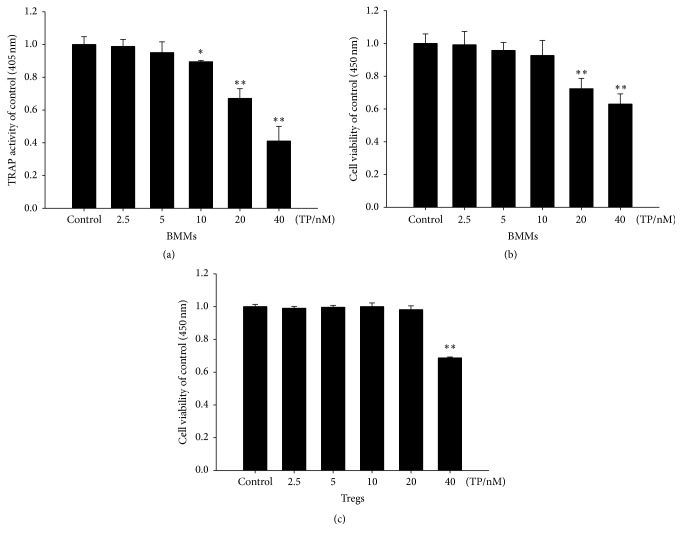
The effect of different concentrations of triptolide on BMMs or Tregs. (a) Mouse BMMs were treated with triptolide (2.5, 5, 10, 20, or 40 nM) in the absence of M-CSF and RANKL. TRAP activity was measured at a wavelength of 405 nm as described above. (b) Mouse BMMs were treated with triptolide (2.5, 5, 10, 20, or 40 nM) in the absence of M-CSF and RANKL. Cell viability was measured at a wavelength of 450 nm using the CCK-8 assay. (c) Activated Tregs were treated with triptolide (2.5, 5, 10, 20, or 40 nM), and cell viability was measured at a wavelength of 450 nm using the CCK-8 assay. ^*∗*^ *P* < 0.05 and ^*∗∗*^ *P* < 0.01 compared with the control group (TP 0 nM group).

Treatment with triptolide resulted in a dose-dependent reduction in both cell viability and TRAP activity. The results showed that triptolide did not affect the viability of Tregs at concentrations ranging from 2.5 nM to 20 nM (Figure 2(c)). We also found that triptolide did not affect the viability of osteoclasts at concentrations ranging from 2.5 nM to 10 nM (Figure 2(b)). After 5 days, TRAP activity was significantly inhibited by triptolide at concentrations ranging from 10 nM to 40 nM (Figure 2(a)). Based on these results, 10 nM triptolide was determined to be the optimal dose.

### 3.5. The Effect of Triptolide on Cocultures of Tregs and BMMs

**Figure 3:**
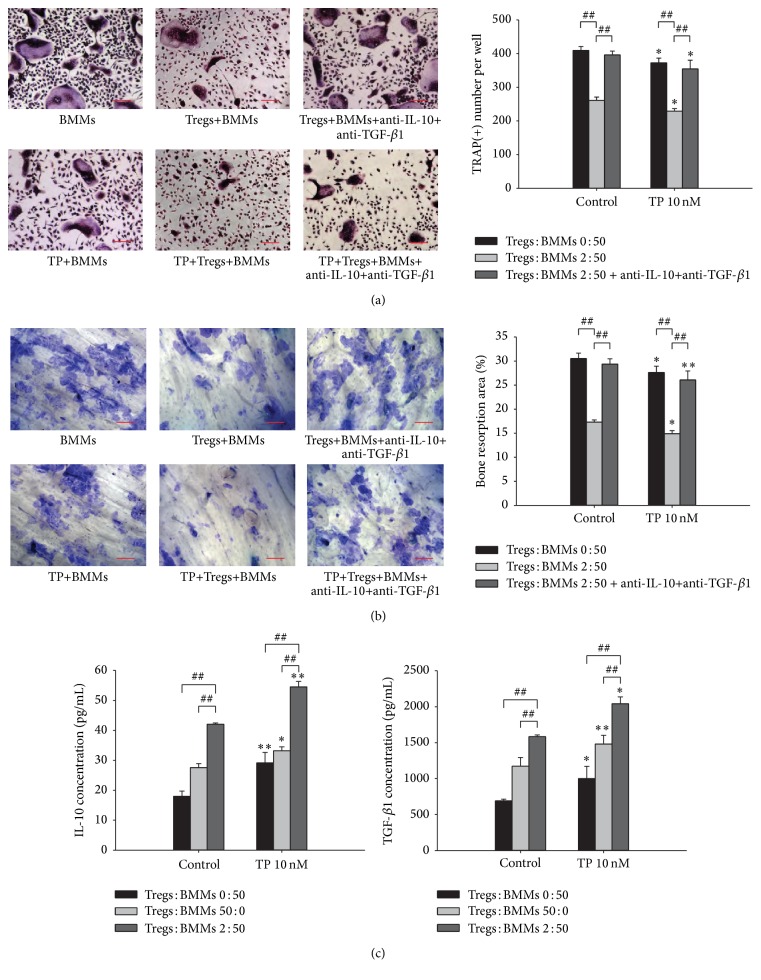
The effect of the indicated concentrations of triptolide on cocultures of Tregs and BMMs. (a) The cells were stained with TRAP, and TRAP-positive cells containing 3 or more nuclei were scored. Scale bars = 100 *μ*m. (b) The area of the bone resorption pits of the osteoclasts was visualized by staining with toluidine blue and measured using an image analysis program. Scale bars = 100 *μ*m. (c) The levels of IL-10 and TGF-*β*1 in the coculture supernatants were measured by ELISA. ^*∗*^ *P* < 0.05 and ^*∗∗*^ *P* < 0.01 compared with the control group (TP 0 nM group). ^#^ *P* < 0.05 and ^##^ *P* < 0.01 compared with the coculture system.

To assess the involvement of Tregs on triptolide-mediated bone metabolism, we added triptolide (10 nM) to a coculture system comprised of Tregs and BMMs at a ratio of 2 : 50. We found that triptolide decreased the number of TRAP-positive MNCs in cultures of BMMs alone as well as in the coculture system (Figure 3(a)). To examine the effects of triptolide on the function of RANKL-induced osteoclasts, we calculated the area of the resorption pits. Triptolide inhibited osteoclast bone resorption in cultures of BMMs alone and in the coculture system. Compared with the cultures of BMMs alone, the coculture system showed a more significant reduction in the number of TRAP-positive MNCs and in the bone resorption area (Figure 3(b)). Collectively, these results clearly demonstrate that the suppressive effect of triptolide is enhanced by the presence of Tregs in the coculture system. To define the molecular mechanism by which triptolide regulated osteoclast differentiation and bone resorption in the presence of Tregs, we detected the expression of IL-10 and TGF-*β*1 released in the cocultures of Tregs and BMMs treated with triptolide and found that they were both higher than those observed in the cultures of BMMs or Tregs alone (Figure 3(c)). To demonstrate that IL-10 and TGF-*β*1 are the key factors responsible for the effects of Tregs on OC differentiation and bone resorption, we treated the coculture systems with anti-IL-10 and anti-TGF-*β*1 neutralizing antibodies (Figures 3(a) and 3(b)). We found that the inhibitory effects of Tregs on OC differentiation and bone resorption could be partially blocked. As shown in Figure 3, compared with Tregs+BMMs, both the number of osteoclasts and bone resorption area were increased significantly after adding anti-IL-10 and anti-TGF-*β*1 blocking antibody. Compared with TP+Tregs+BMMs, both the number of osteoclasts and bone resorption area were increased significantly after adding anti-IL-10 and anti-TGF-*β*1 blocking antibody. So it leads to the conclusion that Tregs inhibit osteoclast differentiation and resorption in IL-10 and TGF-*β*1-dependent manner.

## 4. Discussion

This study was conducted to investigate the effect of triptolide on Treg-mediated osteoclastogenesis and the levels of the inflammatory cytokines IL-10 and TGF-*β*1. We demonstrated the inhibiting effect of triptolide on osteoclast differentiation and bone resorption and showed that the mechanism may involve the promotion of IL-10 and TGF-*β*1 secretion by Tregs. Our previous studies reported that triptolide lowers arthritis scores, delays the onset of collagen-induced arthritis, and increases the levels of TGF-*β* [14]. The disease progression of RA often involves the destruction of bone surrounding the inflamed joints. Thus preventing bone destruction is critical for the treatment of RA [6]. Many studies have indicated that the altered bone remodeling performed by osteoclasts and osteoblasts is the primary cause of bone destruction [15]. Because osteoclasts are important in bone destruction in RA, suppression of osteoclast differentiation and function has become an critical therapeutic target [16]. Accumulating evidences have proven that the skeletal and immune systems are closely associated through cellular and molecular interactions [17].

CD4^+^CD25^+^Foxp3^+^ Tregs are a subset of CD4^+^ T lymphocytes that express the specific transcription factor fork head box P3 (Foxp3). Tregs are instrumental in the maintenance of immune tolerance as well as in the prevention of autoimmunity [18]. It has been shown that Tregs have an anti-inflammatory role in RA patients by its regulatory function [19]. They consist of thymus-derived natural Tregs (nTregs), Tregs induced* in vitro* by IL-2 and TGF-*β* (iTregs), and Tregs induced* in vivo* [20]. Tregs with immunosuppressive activity play an important role in bone homeostasis. An imbalance between T-helper-cell subsets and Tregs contributes to the pathogenesis of osteoporosis [21]. Here we provided the same evidence that Tregs can be induced from CD4^+^CD25^−^ T cells through the activity of TGF-*β* and IL-2 and that they can be activated by anti-CD3 and anti-CD28 [22, 23]. In our study, the CD4^+^CD25^+^ populations were 98.81% pure, and the CD25^+^Foxp3^+^ cells were 96.49% pure. Because the purity of the characteristic surface markers was greater than 95%, we identified the cells as CD4^+^CD25^+^Foxp3^+^ Tregs. Our results showed that coculture of Tregs with BMMs leads to a decrease in both the number of osteoclasts and the area of bone resorption compared to cultures of BMMs alone. To determine whether Treg-mediated inhibition of osteoclasts requires cell-contact, we cocultured Tregs and osteoclast with or without Transwell inserts. We found that the inductions of osteoclast were both reduced, and there was no significant difference between two coculture systems, indicating that the inhibitory mechanism is not dependent on direct cell contact, but that it is dependent on the secretion of two important immunosuppressive cytokines IL-10 and TGF-*β*1. IL-10 is essential for Treg-mediated immune tolerance and functions as a regulatory cytokine that inhibits both antigen presentation and proinflammatory cytokine secretion. Li et al. found that IL-10 could modulate apoptosis in Tregs and control the autoimmune reaction in RA patients [24]. Luo et al. reported that the Tregs isolated from PBMCs could prevent osteoclast differentiation and bone resorption via IL-10 and TGF-*β*1 and that estrogen enhanced this suppressive effect [12]. Li et al. cocultured CD4^+^CD25^−^ T lymphocytes with bone marrow mesenchymal stem cells transfected with TGF-*β*1 and found that TGF-*β*1 inhibits tissue engineered cartilage absorption by inducing the generation of Tregs [25].

Previous studies have shown that triptolide has an effect on CD4^+^ and CD8^+^ cells in Peyer's patch and might be associated with immunosuppressive activities [26]. Triptolide plays a pivotal role in the inhibition of bone resorption by acting on osteoclasts and decreasing their resorption [27]. Wang et al. also revealed that triptolide can enhance the levels of IL-10 in CIA rats and decrease the expression of T cell receptor variable gene mRNA [28]. Zhang et al. reported that triptolide can promote the expansion of CD4^+^CD25^+^ Tregs and increase the levels of IL-10 [29]. In this study, the BMMs and Tregs were separately treated with different concentrations of triptolide (0, 2.5, 5, 10, 20, or 40 nM). There was no statistically significant difference in BMMs viability at the lower concentrations of triptolide (2.5, 5, and 10 nM) compared with the control group. Treatment with 40 nM triptolide induced a significant cytotoxic effect in Tregs. Additionally, the toxicity induced by triptolide was concentration dependent. 10 nM triptolide was chosen in this experiment. In BMMs and Treg cocultures treated with triptolide, both osteoclast differentiation and bone resorption were inhibited more efficiently than in cocultures without triptolide and in BMMs monocultures. After the addition of triptolide, the concentration of IL-10 and TGF-*β*1 in both cocultures and Treg monocultures increased in comparison with BMMs monocultures. Thus, triptolide may enhance the suppressive effect of Tregs on osteoclast differentiation and bone resorption by increasing IL-10 and TGF-*β*1 secretion. Based on these reports, we hypothesized that IL-10 and TGF-*β*1 were the cytokines responsible for the effect of Tregs on osteoclastogenesis. When we treated these cells with both anti-IL-10 and anti-TGF-*β*1 neutralizing antibodies, the osteoclast count and bone resorption areas were completely restored to the levels observed in BMMs monocultures, and the inhibition of triptolide to osteoclast differentiation and bone resorption have also been reduced significantly. This suggests that IL-10 and TGF-*β*1 might be the crucial cytokines through which Tregs mediate osteoclastogenesis. However, there are still some limitations in our experiments; further studies are needed to assess this mechanism using collage-induced arthritis mice* in vivo*. However, our report has significance in that it reveals the possibility that Tregs are involved in the prevention of bone destruction by triptolide in RA.

## 5. Conclusions

Together, we showed that Tregs can inhibit osteoclast differentiation and reduce the resorption of osteoclasts and demonstrated that the mechanism of this activity was mediated by the secretion of IL-10 and TGF-*β*1. Furthermore, we found that triptolide enhanced the level of IL-10 and TGF-*β*1 secreted by Tregs, which further inhibited osteoclast formation and bone resorption. Therefore, Tregs are likely involved in the triptolide-mediated regulation of bone metabolism and act as potent suppressors of osteoclastogenesis via the secretion of IL-10 and TGF-*β*1. Although many checkpoints remain to be passed, these results may indicate a novel mechanism for triptolide in the retardation of the bone destruction observed in RA.
